# Integrated transcriptomics reveals master regulators of lung adenocarcinoma and novel repositioning of drug candidates

**DOI:** 10.1002/cam4.2493

**Published:** 2019-09-10

**Authors:** Marco Antônio De Bastiani, Fábio Klamt

**Affiliations:** ^1^ Laboratory of Cellular Biochemistry Department of Biochemistry Federal University of Rio Grande do Sul (UFRGS) Porto Alegre RS Brazil; ^2^ National Institute of Science and Technology for Translational Medicine (INCT‐TM) Porto Alegre RS Brazil

**Keywords:** computational drug repositioning, connectivity map, lung cancer, master regulator, transcriptomic

## Abstract

**Background:**

Lung adenocarcinoma is the major cause of cancer‐related deaths in the world. Given this, the importance of research on its pathophysiology and therapy remains a key health issue. To assist in this endeavor, recent oncology studies are adopting Systems Biology approaches and bioinformatics to analyze and understand *omics* data, bringing new insights about this disease and its treatment.

**Methods:**

We used reverse engineering of transcriptomic data to reconstruct nontumorous lung reference networks, focusing on transcription factors (TFs) and their inferred target genes, referred as regulatory units or regulons. Afterwards, we used 13 case‐control studies to identify TFs acting as master regulators of the disease and their regulatory units. Furthermore, the inferred activation patterns of regulons were used to evaluate patient *survival* and search drug candidates for repositioning.

**Results:**

The regulatory units under the influence of *ATOH8*, *DACH1*, *EPAS1*, *ETV5*, *FOXA2*, *FOXM1*, *HOXA4*, *SMAD6*, and *UHRF1* transcription factors were consistently associated with the pathological phenotype, suggesting that they may be master regulators of lung adenocarcinoma. We also observed that the inferred activity of *FOXA2*, *FOXM1*, and *UHRF1* was significantly associated with risk of death in patients. Finally, we obtained deptropine, promazine, valproic acid, azacyclonol, methotrexate, and ChemBridge ID compound 5109870 as potential candidates to revert the molecular profile leading to decreased survival.

**Conclusion:**

Using an integrated transcriptomics approach, we identified master regulator candidates involved with the development and prognostic of lung adenocarcinoma, as well as potential drugs for repurposing.

## Graphical abstract

This study used an integrated transcriptomics approach to investigate transcription factors acting as master regulators of lung adenocarcinoma and assessed their potential prognostic value. This strategy retrieved nine master regulator candidates, three of them significantly associated with patient survival. Finally, these master regulators with prognostic value were used to query drug candidates for repositioning, retrieving six molecules able to revert the molecular profile leading to the pathological state.

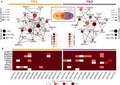

## 1 INTRODUCTION

Historically, lung cancer is one of the most insidious and lethal menaces of oncology, remaining the major cause of cancer mortality among men and women for the past decades.^1^ The two main types of lung cancer are small cell lung cancer (SCLC) and nonsmall cell lung cancer (NSCLC), the latter accounting for ~85% of diagnosed cases.^2^ NSCLC collectively includes numerous epithelial‐derived tumors, of which adenocarcinoma and squamous cell carcinoma are the most frequent. Moreover, lung adenocarcinoma is the most common subtype in never smokers and its incidence is increasing steadily in the United States, Europe, and East Asia, including Japan.^3^, ^4^ Appropriate identification of histologic types is important because it affects prognosis, therapy selection, and considerations for molecular testing.^5^ Therefore, type‐specific molecular research is a reasonable standpoint to translate the biological diversity of lung cancer into clinically relevant subpopulation selection and subsequent customized therapy guidelines.

Although the search and study of single genes associated with cancer have been a commonplace in oncology research for many years, recent systems biology views emerged to introduce new complexity and understanding to the scenario.^6^ In this context, methods evaluating RNA expression levels, such as microarray and RNA‐seq, have become valuable instruments to assess the dynamic properties of biological systems in a fast, broad, and reliable way. Besides the technological aspect, the development of bioinformatics analytical methods and tools has also aided enormously our capacity to extract knowledge from *omics* technologies.^7^

One important area of systems biology involves the identification and understanding of gene regulatory networks (GRNs). For this, a commonly used approach is the so‐called reverse engineering, a process of revealing the network structure of a biological system by reasoning backward from observed data.^8^ The concept of master regulators (MRs) is inserted into the evaluation of GRNs and used to describe elements situated in higher positions of the biological network hierarchy, participating in the specification of cellular lineages by regulating multiple network elements, ultimately controlling the ability to specify/respecify cellular outcome.^9^ In accordance with this concept of MR by default, we have the physiological role of transcription factors (TFs), which enable a relatively small number of molecules to generate a large diversity of cell types and phenotypic states.^10^, ^11^

Additionally, following the momentum of big data basic research, clinical and translational communities are also incorporating this paradigm into their view of patient treatment and therapy. Recently, pharmacology research is attempting to break the “one disease, one target, one drug” model of drug discovery through the incorporation of a new archetype of drug research, namely systems pharmacology.^12^, ^13^ This approach merges systems view, drug repositioning, and bioinformatics to explore the possibility that drugs can alter the expression profiles of pathological network modules of complex biological systems. In a network, modules represent highly interconnected local regions, such as biochemical pathways for example.^6^ Accordingly, modules‐based drug repositioning has been proposed to retrieve drug candidates in breast cancer^14^ and colorectal adenocarcinoma.^15^ Both these approaches employed the molecular Connectivity Map (CMap) strategy of drug repurposing, which recognizes that biological elements have several interdependencies and that attempts to disregard such a notion using single‐targeted interventions are probably futile. Thus, the proposition is to modify the entire state of the pathological system toward the physiological scenario, through modulation of many targets simultaneously.^16^

In this study, 13 transcriptomic datasets were employed to identify potential MRs of lung adenocarcinoma using the ARACNe algorithm and GRNs centered on TFs and their targets. The inferred targets of nine MR candidates—*ATOH8*, *DACH1*, *EPAS1*, *ETV5*, *FOXA2*, *FOXM1*, *HOXA4*, *SMAD6*, and *UHRF1*—were consistently enriched with differentially expressed genes overrepresented in the pathological phenotype. Furthermore, the inferred activity of *FOXA2*, *FOXM1*, and *UHRF1* were significantly associated with survival in several lung adenocarcinoma cohorts. Finally, we used a module‐oriented CMap approach to query drugs able to revert the pathological gene expression profiles of these regulatory units. This strategy retrieved six repositioning candidate molecules—deptropine, promazine, valproic acid, azacyclonol, methotrexate, and ChemBridge ID compound 5109870.

## 2 METHODS

### 2.1 High‐throughput data acquisition

All expression profiles used in this study were obtained from Gene Expression Omnibus (GEO) database using the GEOquery package (version 2.48.0).^17^ Available processed expression data were transformed to logarithmic scale prior to further analyses, when necessary. Two nontumorous lung datasets were used to infer the reference transcriptional networks centered on transcription factors. Thirteen datasets of case‐control (cancer vs unaffected lung tissue) were acquired to compute altered regulatory units based on the reference networks. Finally, 11 different datasets of lung adenocarcinoma were employed to evaluate the survival associations of patients based on altered regulatory units. Details and summary descriptions of the expression data acquired are presented in Table 1.

**Table 1:** Gene expression datasets employed to retrieve master regulators of lung adenocarcinoma

Purpose	GEOID	Sample Size	Method
Nontumorous lung transcriptional network reconstruction (TN1)	GSE23546	904 (Laval+UBC)	Microarray
Nontumorous lung transcriptional network reconstruction (TN2)	GSE71181	284	Microarray
Master regulator inferences (case vs control studies)	GSE10072	104	Microarray
	GSE11969	95	Microarray
	GSE21933	32	Microarray
	GSE27262	50	Microarray
	GSE31552	47	Microarray
	GSE32665	179	Microarray
	GSE32863	116	Microarray
	GSE40275	84	Microarray
	GSE43458	110	Microarray
	GSE62113	25	Microarray
	GSE74706	28	Microarray
	GSE102511	31	RNA‐seq
	GSE87340	27	RNA‐seq
Survival	GSE14814	71	Microarray
	GSE26939	116	Microarray
	GSE29013	55	Microarray
	GSE11969	95	Microarray
	GSE37745	106	Microarray
	GSE41271	178	Microarray
	GSE42127	132	Microarray
	GSE50081	127	Microarray
	GSE87340	27	RNA‐seq
	TCGA	594	RNA‐seq

### 2.2 Reference transcriptional network inference

The transcriptional networks (TNs) centered on TFs and their predicted target genes were inferred using nontumorous lung tissue. Herein, the reference transcriptional network inferred using GSE23546 will be referred to as TN1 and the one using GSE71181 as TN2 (Table 1). In this study, the term *regulatory unit* or *regulon* is used to describe the groups of inferred genes and their associated TFs. Transcriptional networks were computed using the RTN package (version 2.4.6)^18^, ^19^ employing mutual information‐based Algorithm for the Reconstruction of Accurate Cellular Networks (ARACNe) method.^20^ Interactions were evaluated using a MI threshold cutoff by permutation and by bootstrap, creating a consensus network. Additionally, a final step employs a Data Processing Inequality algorithm with null tolerance to eliminate associations likely mediated by another TF. Our network reconstruction parameters were 1000 permutations, with a *P*‐value cutoff of .001, and 100 bootstraps (all remaining parameter were kept default).

### 2.3 Master regulator inference and two‐tailed gene set enrichment analysis

For the MR inference, we employed the master regulator analysis (MRA) described by Carro and colleagues 21 to the regulatory units of each reference TN. The algorithm computes the statistical overrepresentation (enrichment) of differentially expressed genes (DEG), obtained from differential expression analysis, in the regulatory units of TN1 and TN2. Our criteria of differential expression were false discovery rate (FDR)‐adjusted *P*‐value <.05 and absolute log fold change (logFC) > 1. Regulons with statistical enrichment of DEG and with >50 elements (regulon size >50), in ≥80% of the queried case‐control studies, were considered altered in the lung adenocarcinoma, thus considered potential master regulators of the disease.

Two‐tailed GSEA was also performed using the RTN package (version 2.4.6), with a *P*‐value cutoff set to .05 and using 1000 permutations. Briefly, Pearson's correlation was used to split the regulatory units into two subgroups: positively associated targets (A) and negatively associated targets (B). Afterward, the phenotype association of each subgroup was tested by the GSEA^22^ statistics, resulting in independent enrichment scores (ES) for each subgroup. An additional step was carried out to test the differential enrichment (ES_A_ − ES_B_), considering that a maximum deviation from zero near opposite extremes and a good separation of the two distributions are desirable for a clear association. Thus, a high negative differential score implies that the regulon is repressed in the disorder phenotype, while a high positive differential score indicates that the regulon is induced in the disorder phenotype.

The Bioconductor (version 3.7) package limma (version 3.36.5)^17^ was employed to compute differential expression analysis of case‐control studies and obtain DEG and logFC for both MRA and GSEA inputs. Importantly, all analyses were implemented equally and independently to all case‐control studies employed against each TN reference.

### 2.4 Survival analyses

Multivariable Cox proportional hazards regressions were performed using regulon's ES, adjusted for clinical variables according to available information. Diagnostic analyses of the models were performed based on Martingale and weighted Schoenfeld residuals plots and tests^23^; clinical variables available were stratified to meet the proportional hazards assumption when necessary. The ES of queried regulatory units were firstly assessed in the studies selected for survival analyses (Table 1) through gene set variation analysis (GSVA) method.^24^ This gene set enrichment method estimates group gene activity variation over a sample population in an unsupervised manner and provides the scores used to assess the influence of regulons’ activity over the risk of death in patients. These scores were used to compute survival models for all selected studies in both TN references. Additionally, enrichment scores from all studies in a given TN reference were z‐score standardized, merged and evaluated together for their association with risk. Finally, Kaplan‐Meier curves were constructed by discretizing of ES distributions into three segments (low = lower quartile; mid = IQR; high = upper quartile). Risk differences between groups were assessed using the log‐rank test.

Similarly, for gene expression association with survival risk, we employed multivariable Cox proportional hazards regression using expression data of each selected study in both TN reference contexts.

Cox models and Kaplan‐Meier curves were computed and graphed using survival (version 2.43‐3) and/or survminer (version 0.4.3) packages.^25^, ^26^ Importantly, all analyses were implemented equally and independently to all survival studies employed against each TN reference.

### 2.5 Computational drug repositioning using connectivity map

Altered regulatory units in lung adenocarcinoma were queried for drug candidates capable of reverting their expression profiles using a module‐oriented adaptation of classical connectivity maps (CMap) method of drug repositioning.^27^, ^28^ Briefly, for each case‐control study in both TN references, inferred genes of several regulons were selected, combined, and their logFC values submitted to the CMap analysis workflow using PharmacoGx package (version 1.10.3).^29^

### 2.6 Statistical software

All analyses were computed in R statistical environment (version 3.5.2),^30^ employing the above‐mentioned packages for each analysis. Networks were computed using RTN package and network figures were constructed using RedeR package (version 1.28.0).^31^ Other plots were created with ggplot2 package (version 3.1.0).^32^

## 3 RESULTS

### 3.1 Human lung regulatory network reconstruction

Two large public transcriptional datasets of nontumorous lung tissue obtained from GEO were used for the regulatory network reconstruction. Our approach assumes that transcription factors’ activities are valuable agents of phenotypic difference or modulation, and that their effects can be assessed through fluctuations in the expression of their target genes. Thus, the goal of the network reconstruction is to infer target genes of TFs through their coassociation and encapsulate them into lists of genes, which will be addressed as *regulatory units* or *regulons*.

The resulted reference networks, herein named TN1 and TN2, derived from GSE23546 and GSE71181, respectively, constitute the starting inquiry point of our analyses. TN1 represents a superseries of 904 nontumorous lung samples, whereas TN2 represents 284 noninvolved lung parenchyma from adenocarcinoma patients. Transcripts were classified as transcription factors when annotated in the Gene Ontology with the identifier GO:0003700 (transcription factor activity, sequence‐specific DNA binding). Following the networks’ reconstruction, all subsequent analyses were implemented over both TN or their intersected results. Figure S1 summarizes the workflow employed in our study for master regulators of lung adenocarcinoma, their influence in patient survival and their potential to identify drug repositioning candidates.

### 3.2 Inference and activity of lung adenocarcinoma master regulators

**Figure 1:**
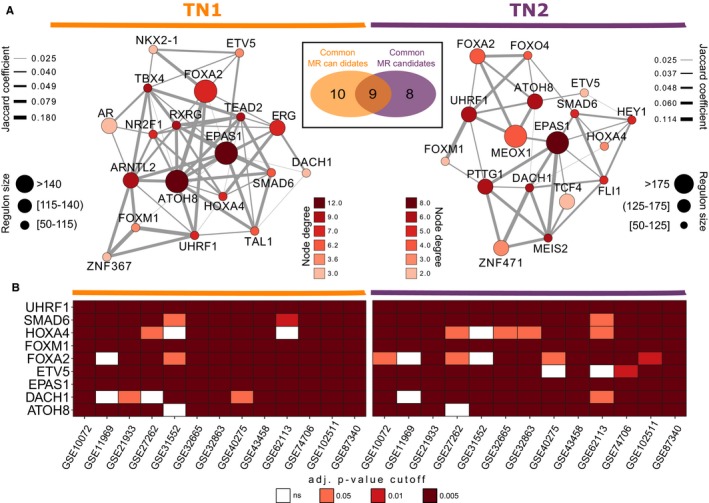
*Common* and *Consensus* Master Regulator of Lung Adenocarcinoma. A, Subnetwork of inferred *common* master regulators and their association for each reference transcription network. Node sizes map the number of genes inferred for a given transcription factor, the number of connections (degree) was mapped to the color of the nodes and edge widths represent the number of overlapped genes shared by pairs of TF *Insert* shows a Venn diagram of the regulons in TN1 and TN2 networks (intersection regulons were termed *consensus* master regulators). B, Master regulator analysis showing the statistical overrepresentation (enrichment) of differentially expressed genes in each *consensus* master regulator, for all case‐control datasets and for both reference TN. Our criteria of differential expression were false discovery rate (FDR)‐adjusted *P*‐value <.05 and absolute log fold change (logFC) > 1

Nontumorous lung network reconstruction yielded sets of regulatory units centered on TFs for each TN as outputs. We next questioned which of these regulatory units were altered in lung adenocarcinoma. To address this issue, we collected 13 different transcriptomic datasets containing tumoral and nontumoral tissue information and used the MRA method proposed by Carro and collaborators^21^ to infer consistently altered regulons. We found 19 regulons significantly enriched with differentially expressed genes (FDR‐adjusted *P*‐value <.05 and absolute logFC > 1) in ≥80% of the 13 studies for TN1 and 17 regulons for TN2. We termed the transcription factors modulating these regulatory units *common* MR of the disease. Figure 1A shows the subnetwork representation of these impaired regulons and their associations with each other in both TNs. Table S4 shows additional information about the subnetwork structures, as well as TN1 and TN2 reconstruction overlaps. Additionally, we observed nine *common* MRs consistently dysregulated in both reference TN (Figure 1A *insert*), which were referred to as *consensus* MR. These *consensus* MRs were the regulons under the influence of ATOH8, DACH1, EPAS1, ETV5, FOXA2, FOXM1, HOXA4, SMAD6, and UHRF1 transcription factors (Figure 1B).

**Figure 2:**
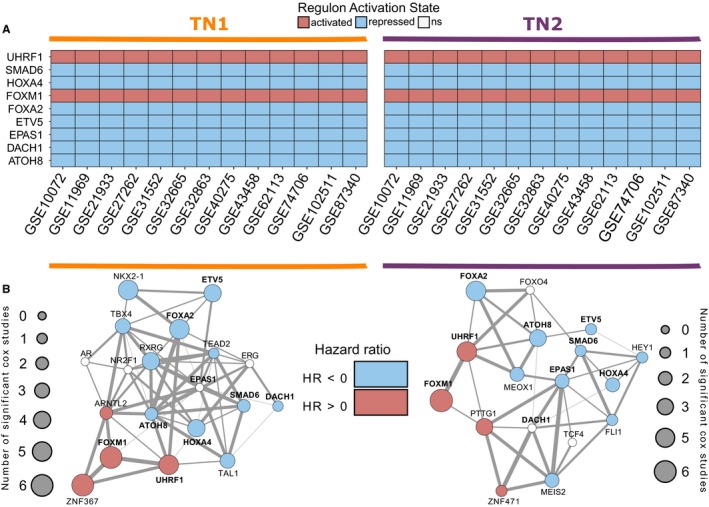
Activation State and Survival Network of *Consensus* Master Regulators. A, Two‐tailed gene set enrichment analysis was used to query the activation state of *consensus* master regulators in both reference TNs. B, Regulon enrichment was used to investigate the altered regulatory units’ association with survival risk using Cox proportional hazards regression in 10 transcriptomic studies. The results were mapped over the *common* MR networks in each transcription network. Node size represents the number of studies in which a significant association of regulons’ activity with survival was observed. Node color shows the hazard ratio of these associations

For each regulatory unit in both TNs, the network reconstruction also infers the transcription factors' modes of action on targets through their expression correlation. With this information, we queried the state of activation of the *consensus* MRs in TN1 and TN2 using a two‐tailed variation of the GSEA method. Figure 2A shows the inferred activation states observed for the nine MR candidates in both TNs. We observed repression of six MRs—*ATOH8*, *DACH1*, *EPAS1*, *ETV5*, *FOXA2*, *HOXA4,* and *SMAD6*—meaning that the negative targets of these transcription factors are upregulated in the disease phenotype, whereas their positive targets are downregulated (FDR‐adjusted *P*‐value <.05). On the other hand, *FOXM1* and *UHRF1* were activated in lung adenocarcinoma, with increased expression of their positive targets and/or decreased expression of their negative targets.

### 3.3 Master regulators of survival in lung adenocarcinoma

Besides investigating the association of transcription factors and their regulatory units with the diseased phenotype by identifying master regulator candidates, we asked how variations in the activity state of these regulons would affect patient outcome. To answer this, we used multiple Cox proportional hazards analyses of 10 different datasets of transcriptomic information (Table 1). Regulon activity was estimated in an unsupervised manner through gene set variant analysis (GSVA), providing ES of each patient with which survival models were fit.

Firstly, we modeled the influence of each *common* MR enrichment score on patient risk in all 10 studies from both reference TNs. Figure 2B shows the count result of these analyses in a survival network. Node size maps the number of transcriptomic datasets in which the *common* MR enrichment score showed significant association with patient risk, adjusted for the clinical variables available in each dataset. Node colors represent the direction of the hazard ratio (HR) for the studies showing significant survival risk. HR >0 (pink nodes) means that increased ES (regulon activation) is associated with an increase in patient risk of death, whereas HR <0 (blue nodes) means that increased ES is associated with a decrease in patient risk of death. White nodes map the regulatory units which showed no significant association with survival, or inconsistency in HR direction.

**Figure 3:**
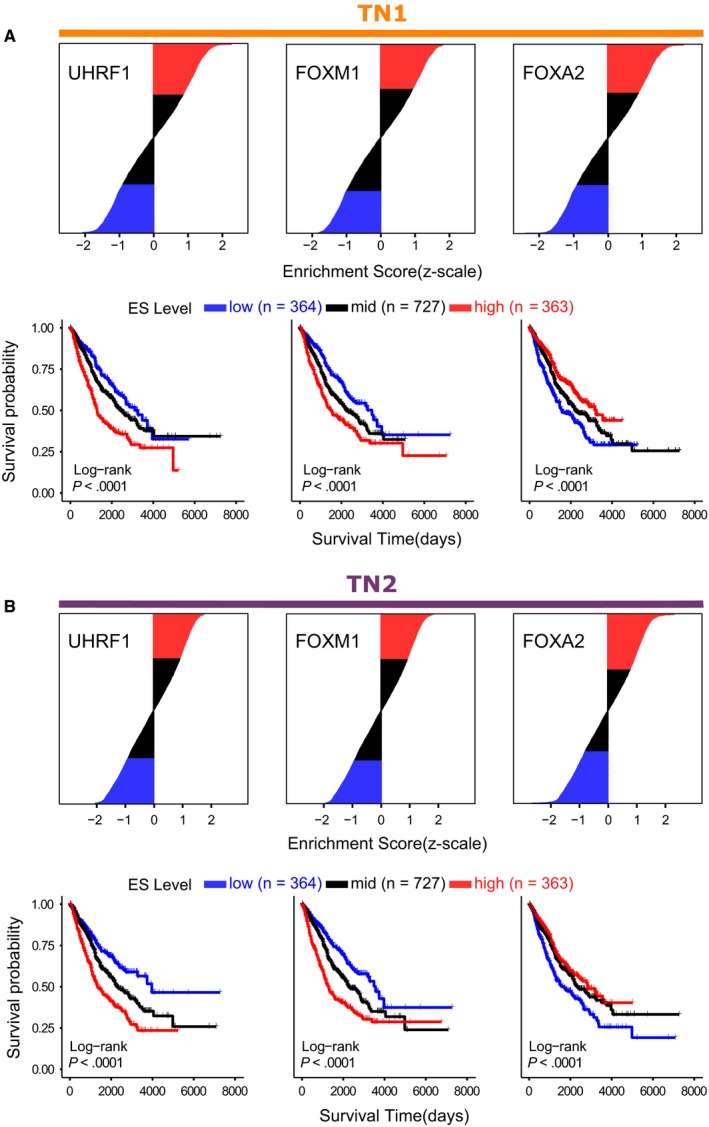
Kaplan‐Meier Survival Curves of Master Regulators. Enrichment scores of *consensus* master regulators consistently associated with patient survival were standardized (z‐score), merged and their distributions were discretized. The discretized quartile segments (low = first quartile; mid = second quartile; high = third quartile) were then evaluated using Kaplan‐Meier curves and log‐rank test (lower segments) in both (A) TN1 and (B) TN2 reference networks

Interestingly, we observed three *consensus* MRs—*UHRF1*, *FOXM1*, and *FOXA2*—significantly associated with survival in ≥50% of the studies queried, for both reference TN (Figure S3). Next, we evaluated the z‐scaled, combined ES distribution of these regulatory units using Kaplan‐Meier survival curves (Figure 3). We observed that the discretized, standardized ES distributions of all three MRs, especially in the first and third quartiles, significantly separate patient risk of death (log‐rank *P*‐value <.001). Additionally, to decompose the net influence of each target gene in the regulons, we investigated the importance of their expression to patient survival in all three consensus MRs (Figure S4). Indeed, we can observe an overrepresentation of targets whose expressions are significantly associated with risk of death in ≥50% of the datasets investigated, including proliferation markers such as the marker of proliferation Ki‐67 (*MKI67*) and checkpoint kinase 1 (*CHEK1*). Table S1 shows detailed information of each target gene coefficient, adjusted for clinical variables, in all datasets for both reference TNs.

### 3.4 Master regulator connectivity map (MRCMap)

After retrieving several possible MRs of lung adenocarcinoma, their regulatory units, and these units' association with patient risk, we queried repositioning drug candidates that would revert the expression profiles of these regulons using a modular variation of the connectivity map method. We employed three different gene list queries, using all case‐control studies and both reference TNs.

In the first query, we merged the *common* MRs’ regulatory units into a single gene list input. Seventy‐one drugs emerged consistently reverting this gene list expression profile in all 13 connectivity maps of case‐control (connectivity score <0; FDR‐adjusted *P*‐value <.05) studies for TN1; whereas 57 drugs were observed for TN2. Next, the merged regulons of *consensus* MR were used in the same way, resulting in 59 drug candidates for TN1 and 43 for TN2. Finally, querying the list of genes acquired by merging the three regulatory units associated with patient risk yielded 89 drugs for TN1 and 18 drugs for TN2. Table S2 and Table S3 summarize the results obtained using the master regulators connectivity map pipeline for TN1 and TN2, respectively.

**Figure 4:**
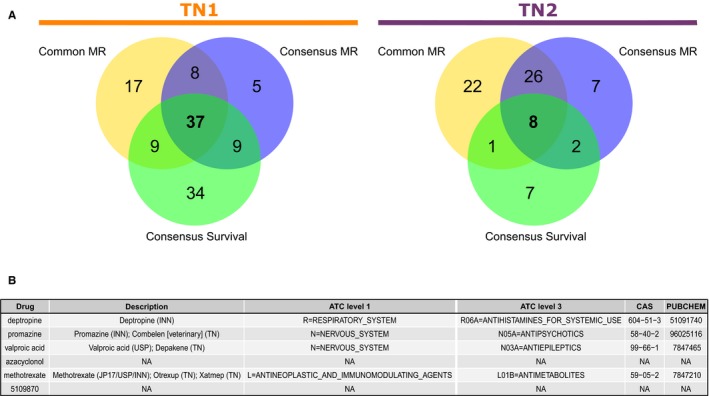
Master Regulator Connectivity Maps. A, Venn diagrams of drug candidates obtained from the connectivity maps method. Genes from the *common* master regulator regulons, the eight *consensus* master regulator regulons, and the three master regulators of survival regulons were each combined to form the different sets of gene lists. We only counted molecules in which connectivity score reverted the expression profiles of each gene list; and had FDR‐adjusted *P*‐value <.05 in all 13 adenocarcinoma case‐control studies. Left diagram shows the count of drug candidates found for TN1 and right diagram shows counts of candidates found for TN2 reference network. B, Table showing the six drugs observed in the intersections of the connectivity maps of both reference TNs, their anatomical therapeutic chemical (ATC) classification, and chemical abstracts service (CAS) registry number

Figure 4A shows the Venn diagrams of the number of drug candidates for repositioning and their intersections, given each gene list input, for both reference TNs. Of these intersections, six molecules (Figure 4B) were consistently present in both reference transcription network MRCMap, namely deptropine, promazine, valproic acid, azacyclonol, methotrexate, and ChemBridge ID compound 5109870 (https://www.hit2lead.com/screening-compounds/5109870).

## 4 DISCUSSION

Molecular aberrations in important genes have been the standard of oncology research for many years, especially when searching for cancer biomarkers. However, we are past the phase of single‐gene paradigms and have entered the era of Big Data and systems thinking. Indeed, systems and network biology expanded our perception of complex diseases beyond the actors of the pathological narrative. In this study, we have used a gene regulatory reconstruction approach centered on TFs to study lung adenocarcinoma and employed a systems pharmacology strategy to prospect drugs for repositioning, observing nine TFs acting as master regulators of the disease, and six drug candidates for repurposing.

We used the term GO:0003700 (transcription factor activity, sequence‐specific DNA binding) to center the regulatory network reconstruction of nontumorous data. Gene Ontology is the leading information hub on biological knowledge about gene and their products across genomic resources. The ontology is expanded as new findings rise and new annotations occur to keep up with literature. However, despite the efforts, at times the association of ontology and terms with genes is incomplete. This inherent incompleteness hinders the evaluation of computational methods and should be acknowledged when using the database.^33^ In our case, this means that some elements under GO:0003700 could include genes not specifically classified as TFs. For example, recently Lambert and collaborators compiled a review on human transcription factors, highlighting the difficulties regarding the identification of TF‐target association and the methods employed to evaluate these interactions. Additionally, they also manually curated the current TF collection, combining putative lists from several sources, including GO. The final count encompassed 1639 known or likely human TFs.^34^ According to their curated list, two of the MR candidates we identified (*SMAD6* and *UHRF1*) are not considered true TFs.

Although the master regulator concept is not exclusive to transcription factors, these considerations are important when evaluating our results. Nevertheless, we believe the approach employed here can bring new hints about the pathological scenario of adenocarcinoma. For example, *ATOH8* (atonal Homolog 8) belongs to a group of basic‐helix‐loop‐helix (bHLH) transcription factors involved in the regulation of cardiovascular development, hematopoiesis, skeletal muscle development, neurogenesis, and embryogenesis.^35^ Dysregulation of this TF was identified with malignant phenotype in several types of cancer.^35^, ^36^ However, its exact role in cancer development remains unclear. In our study, the inferred regulatory unit under *ATOH8* influence was associated with malignant phenotype and repressed in the pathology. To our knowledge, this is the first time *ATOH8* is reported associated with lung adenocarcinoma.

On the other hand, many of the MR candidates obtained by our analyses have been previously reported in lung cancer. For example, *ETV5* (Ets variant 5) is part of the ETS family of transcription factors, which deregulation can alter the expression of proteins involved in stem cell development, cell senescence, proliferation, migration, apoptosis, and tumorigenesis.^37^ In lung adenocarcinoma, *ETV5* was shown to inhibit N‐cadherin‐dependent adhesion in cooperation with its cotranscriptional factor LPP (lipoma‐preferred partner), favoring epithelial‐to‐mesenchymal transition (EMT) and metastastatic potential of NSCLC cell line PC14PE6 in vivo.^38^ On the other hand, loss of *ETV5* seems to impair lung recovery from drug‐induced damage, triggering tumor initiation by oncogenic Kras.^39^ In our study, inferred *ETV5* activity was observed repressed in lung adenocarcinoma using two‐tailed GSEA analysis. Thus, it remains speculative whether these events are tumor specific or depend on cellular‐specific circumstances, and what is the exact role of this transcription factor in this disease.

*SMAD6*, *HOXA2,* and *UHRF1* are also poorly explored potential regulators of NSCLC *SMAD6* negatively regulates BMP, TGF‐beta, and activin signaling pathways, which control growth, differentiation, apoptosis of cells, and angiogenesis.^40^ Hints to its potential tumor‐suppressing activity are reported in several cancers.^41^, ^42^, ^43^ Yet, in lung cancer, Jeon and collaborators initially proposed that *SMAD6* reduction inhibits cancer cell growth and induces apoptosis.^44^ Meanwhile, however, other studies reinforce the suppressor potential observed in other cancers.^45^, ^46^, ^47^ Our findings also support these latter observations, since our analysis indicated that *SMAD6* regulon activity was repressed in the lung adenocarcinoma case‐control studies utilized.

*HOXA4* (Homeobox A4) belongs to the Homeobox gene family of transcription factors associated with cell differentiation and embryonic development control.^48^ *HOXA4* regulation seems to associate with lung cancer cell proliferation, migration, and invasion in vitro and in vivo, besides poor prognosis in patients.^49^, ^50^ We identified that the inferred regulatory unit of *HOXA4* is enriched with differentially expressed genes and its activity was suppressed in tumor tissues compared to control tissues. Therefore, our evaluation also supports the role of *HOXA4* as a potential tumor suppressor in lung adenocarcinoma.

*UHRF1* (Ubiquitin Like with PHD and Ring Finger Domains 1) is essential for cell proliferation and DNA methylation maintenance.^51^ It possibly exerts its effects on expression via epigenetic alteration, by interacting with DNMT1 (cytosine‐5 DNA methyltransferases) and HDAC1 (histone deacetylase 1), thus propagating or maintaining epigenetic patterns. These interplays may lead to tumor initiation, progression, metastasis, and relapse.^51^, ^52^, ^53^ *UHRF1* is reported as a tumor epigenetic modulator toward malignancy in lung cancer and as a diagnostic biomarker.^54^, ^55^, ^56^ Our results are in agreement with this line of evidence, since its inferred activity was upregulated in the two‐tailed GSEA analysis and associated with poor prognosis in survival analyses.

The four remaining transcription factors retrieved as potential MRs—*DACH1*, *EPAS1*, *FOXA2,* and *FOXM1*—have been extensively reported in associations with cancers in literature. *DACH1* is a key member of the retinal determination gene network, a group which affects cell cycle regulation and cancer cell growth, EMT, invasion, and migration in a context‐specific manner.^57^ Its reduced expression is observed in several types of cancer, mostly associated with poor prognosis.^58^ Our results also point that these TFs' inferred activity is repressed in lung adenocarcinoma, similarly to previous lung cancer studies.^59^, ^60^, ^61^ Interestingly, *DACH1* activity did not show a consistently remarkable association with risk in our survival evaluation. This may be because it acts as a regulator of targeted genes also through interaction with other transcription factors.^58^ For example, it can directly bind and enhance several functions of p53 in NSCLC,^59^ whereas it also can antagonize *FOXM1* transcriptional modulation through competitive binding of DNA segments.^62^

*EPAS1* (endothelial periodic acid‐Schiff domain protein 1), also known as HIF2‐alpha, is expressed in type II pneumocytes and pulmonary endothelial cells.^63^ Several studies evaluating the role of *EPAS1* in lung cancer demonstrated that its activity is puzzling and ambiguous.^64^, ^65^ Collectively, the literature suggests inhibition of overexpressed *EPAS1* in lung cancer might have beneficial effects but reductions below a critical threshold favor tumorigenesis and are associated with poor prognosis. In our evaluation of 13 transcriptomic studies, the inferred activity of *EPAS1* was consistently repressed in tumor tissues compared to unaffected lung. Although the exact mechanisms directing the pro‐ or antitumoral role remain elusive, it is likely that genetic polymorphisms and epigenetic modifications of *EPAS1* may lead to varied gene expression and outcomes.^65^, ^66^

The two remaining MRs belong to the forkhead box (FOX) gene superfamily, which is described controlling several important biological processes,^67^ including airway epithelial differentiation and branching.^68^ Several studies observed the tumor suppressor potential of *FOXA2* (forkhead box transcription factor A2) in lung cancer through inhibition of EMT, metastasis, and proliferation.^69^, ^70^, ^71^, ^72^ Accordingly, our study shows that the inferred activity of *FOXA2* is repressed in tumor samples. Moreover, overall survival evaluation of *FOXA2* activity showed that populations with lower activity were significantly associated with higher risk of death. The exact molecular mechanisms associated with *FOXA2* expression suppression are pending, but evidences suggest that epigenetic alterations are mediating this phenomena.^70^ On the other hand, given the complex physiology of TFs, it is hard to dissect the effect of other actors influencing *FOXA2* activity. For example, *NKX2‐1* is another master regulator of pulmonary differentiation downregulated in poorly differentiated lung adenocarcinoma, which interacts with *FOXA1*/*FOXA2* in human cell lines, regulates global Foxa1/Foxa2 binding in murine adenocarcinoma and cooperates to inhibit metastasis.^71^, ^73^ Interestingly, our master regulator analysis also retrieved *NKX2‐1* as a common MR of lung adenocarcinoma, sharing several targets with *FOXA2* in TN1.

*FOXM1* (forkhead box M1) plays an important role in cell proliferation, cycle regulation, and is expressed only in proliferating cells.^74^ In fact, the oncogenic property of its overexpression is well recognized in many types of solid tumors,^75^, ^76^, ^77^ associated with tumor EMT, growth, migration, metastasis, multidrug resistance, and radioresistance.^75^, ^78^, ^79^ Recently, Gentles and collaborators observed that an integrated protein‐protein association network of *FOXM1* is significantly enriched with adversely prognostic genes in a pan‐cancer TCGA study, suggesting that this TF is a major driver of inferior survival regardless of cancer type.^80^ We also identified *FOXM1* as a master regulator of lung adenocarcinoma in all 13 transcriptomic case‐control studies employed, for both reference TNs reconstructed. Furthermore, its inferred activity was also observed associated with poor prognosis.

Beside the search for master regulators of the pathology and their effects on prognosis, we also explored a systems pharmacology, module‐oriented implementation of drug repositioning using the connectivity maps paradigm. Unsurprisingly, this approach retrieved molecules already used or proposed in lung cancer therapy, such as methotrexate and valproic acid. The first drug, methotrexate, is one of the oldest, most common and efficient antineoplastic drugs. It acts by inhibiting dihydrofolate reductase to deplete intracellular tetrahydrofolate, impairing thymidylate production and leading to apoptosis or autophagy of highly proliferative cell populations. Unfortunately, its clinical use is associated with various toxicities.^81^, ^82^ Valproic acid is reported to be a class I histone deacetylase (HDAC) inhibitor commonly used to treat mood disorders and epilepsy. Its anticancer effect has been reported in several cancers in recent years, including lung adenocarcinoma,^83^, ^84^, ^85^ through modulations of epigenetic patterns via HDAC inhibition and gene expression modification. Furthermore, many of the MR candidates retrieved by our analyses have reported or suggested epigenetic mechanisms of modulation, which further supports the study and search for pharmacological epigenetic controllers in cancer therapy research.

The remaining drugs have few to no associations with lung cancer therapy in literature, and occasional suggestions in other types of cancer. For example, a previous CMap study also retrieved azacyclonol as a potential inhibitor of transcription factor activity using lung cancer expression profiles,^86^ albeit using a different querying strategy. Promazine, a phenothiazine derivate antipsychotic, had an antitumoral activity in breast cancer cell lines^87^ and was able to induce apoptosis and tumor growth of diffuse large B‐cell lymphoma in vivo and in vitro^88^ Recently, deptropine, a H1‐histamine and muscarinic receptor antagonist, was reported inhibiting cell viability and mammosphere formation of breast cancer stem cells, but it did not inhibit the self‐renewal capacities of MDA‐MB‐231 cells.^89^ Finally, regarding ChemBridge ID compound 5109870, not much is known about its behavior in biological systems besides its iron chelating effect 90, 91 and this is the first study suggesting its potential as an anticancer drug.

Finally, it is important to highlight one aspect of Systems Biology that is difficult to assess using the many network analyses usually employed, that is, the dynamical changes in association patterns between genes during phenotypic transitions.^92^ Indeed, most reverse‐engineering methods model gene networks as static processes, in which interaction changes among elements in the network are not accounted across different conditions.^93^ To address this issue, recent studies have been working toward the development of such methods using a differential coregulation framework.^92^, ^94^ However, the addition of dynamic approaches is no trivial endeavor and these methods can be hindered by network reconstruction stability problems,^95^, ^96^ since network topology is of critical importance in these scenarios.^95^ This can be especially troublesome in multidataset studies such as ours. Thus, we opted to evaluate each TN independently in our study and to highlight the consistencies between results obtained from each network.

The importance of OMICS technologies and Systems Biology to the adoption of a holistic paradigm of biology is undeniable, enhancing our understanding of intricate organizations and uncovering molecular signatures underlying complex cellular phenotypes. Our computational approach retrieved nine TFs potentially acting as master regulators of lung adenocarcinoma phenotype. Among them, several are already described as actors of tumorigenesis. However, this is the first study implicating *ATOH8* with lung cancer development. Evidently, further experimental studies are required to fully address its role in the pathology. We also identified six drug candidates for repositioning which could revert the pathological transcription profiles resulted from deregulated activity patterns the three MR candidates robustly associated with risk of death—*FOXA2*, *FOXM1*, and *UHRF1*. We believe the thorough evaluation of these molecules in cellular and animal models, besides randomized clinical trials can potentially lead to the development of new therapies and therapeutic strategies for treatment.

## CONFLICT OF INTEREST

The authors declare no potential conflicts of interest.

## AUTHOR CONTRIBUTIONS

Marco Antônio De Bastiani was involved in conceptualization, formal analyses, methodology, project administration, writing, reviewing, and editing original draft. Fábio Klamt was involved in resource and funding acquisition, supervision, review, and editing.

## Data Availability

Datasets used in this study can be accessed via NCBI GEO portal (https://www.ncbi.nlm.nih.gov/geo/). Further intermediate data and codes generated are available from the corresponding author on request.
