# Supplementary material for: Integrated transcriptomics reveals master regulators of lung adenocarcinoma and novel repositioning of drug candidates
Source: Cancer Med. 2019 Sep 10;8(15):6717–29. doi: 10.1002/cam4.2493 (PMC6825976; doi:10.1002/cam4.2493)
Supplement: Supplementary file 1 [file CAM4-8-6717-s001.pdf]

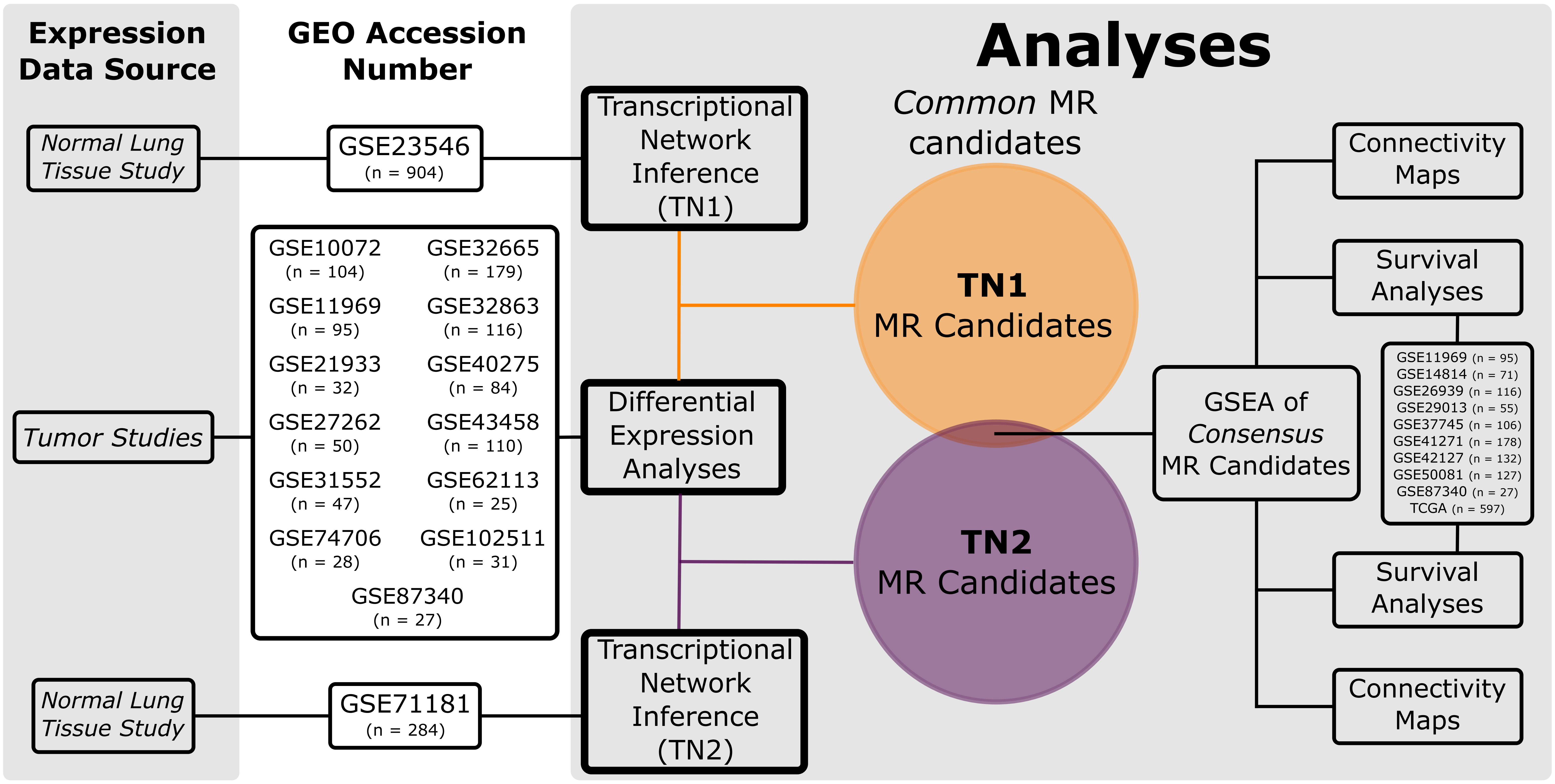
Expression Data Source
GEO Accession Number
Analyses
Normal Lung Tissue Study
GSE23546 (n = 904)
Transcriptional Network Inference (TN1)
Common MR candidates
TN1 MR Candidates
Connectivity Maps
Survival Analyses
Tumor Studies
GSE10072 (n = 104)
GSE32665 (n = 179)
GSE11969 (n = 95)
GSE32863 (n = 116)
GSE21933 (n = 32)
GSE40275 (n = 84)
GSE27262 (n = 50)
GSE43458 (n = 110)
GSE31552 (n = 47)
GSE62113 (n = 25)
GSE74706 (n = 28)
GSE102511 (n = 31)
GSE87340 (n = 27)
Differential Expression Analyses
GSEA of Consensus MR Candidates
GSE11969 (n = 95)
GSE14814 (n = 71)
GSE26939 (n = 116)
GSE29013 (n = 55)
GSE37745 (n = 106)
GSE41271 (n = 178)
GSE42127 (n = 132)
GSE50081 (n = 127)
GSE87340 (n = 27)
TCGA (n = 597)
TN2 MR Candidates
Survival Analyses
Connectivity Maps
Normal Lung Tissue Study
GSE71181 (n = 284)
Transcriptional Network Inference (TN2)
