# Supplementary figures and images for: Integrated transcriptomics reveals master regulators of lung adenocarcinoma and novel repositioning of drug candidates
Source: Cancer Med. 2019 Sep 10;8(15):6717–29. doi: 10.1002/cam4.2493 (PMC6825976; doi:10.1002/cam4.2493)

A

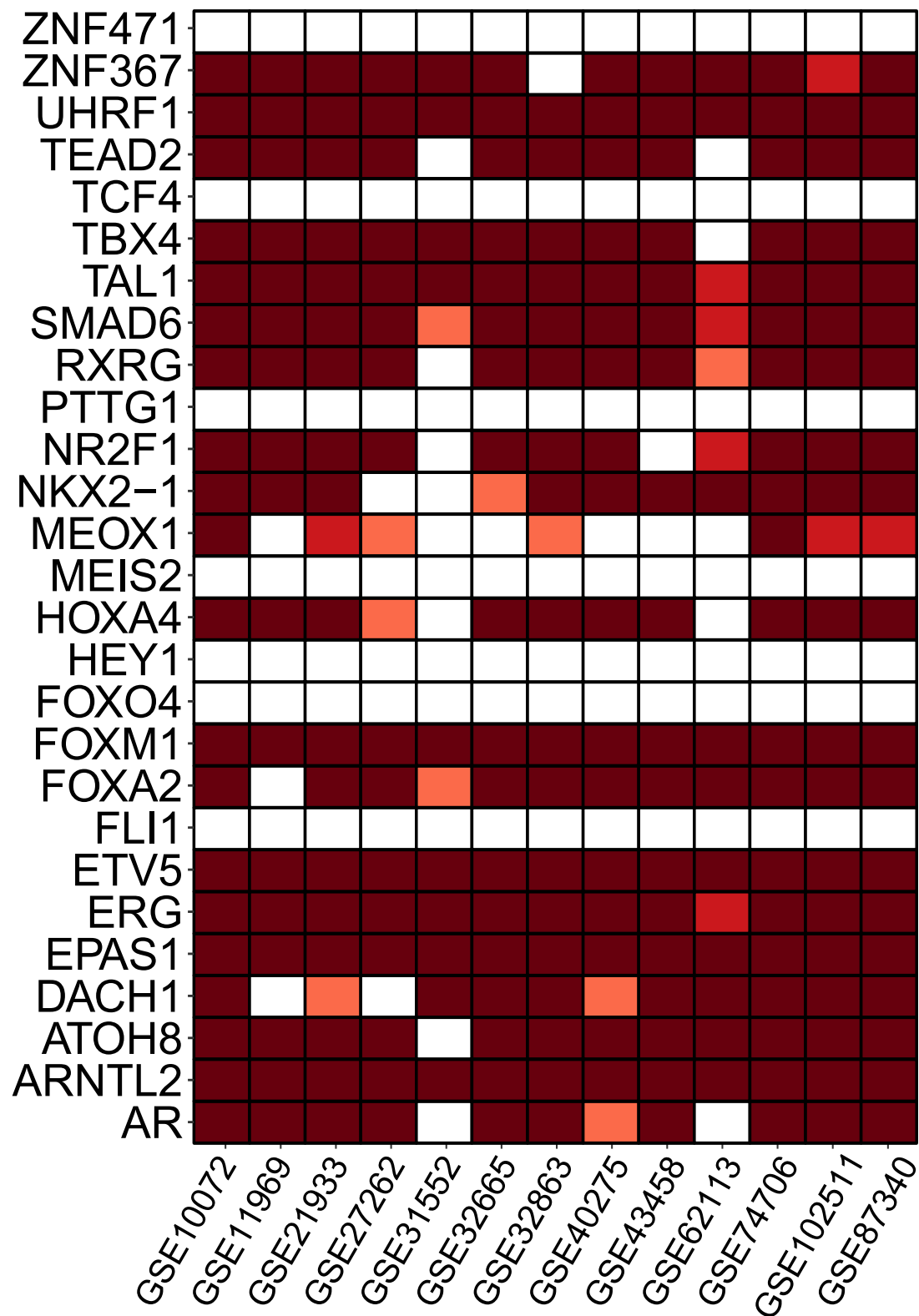

TN1

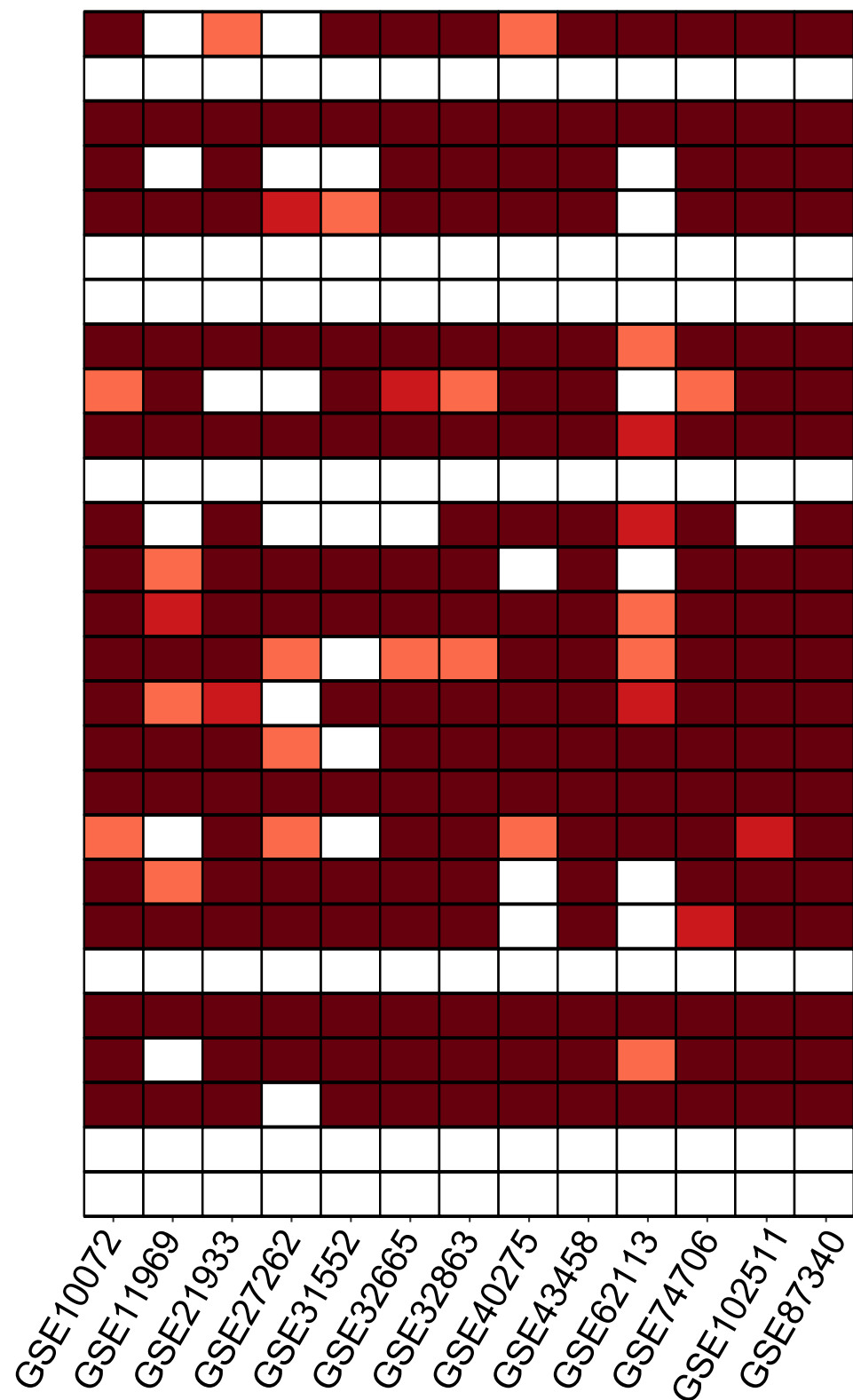

TN2

adj. p-value cutoff

|    |      |      |       |
|----|------|------|-------|
| ns | 0.05 | 0.01 | 0.005 |
|----|------|------|-------|

Supplement: Supplementary file 2 [file CAM4-8-6717-s002.pdf]

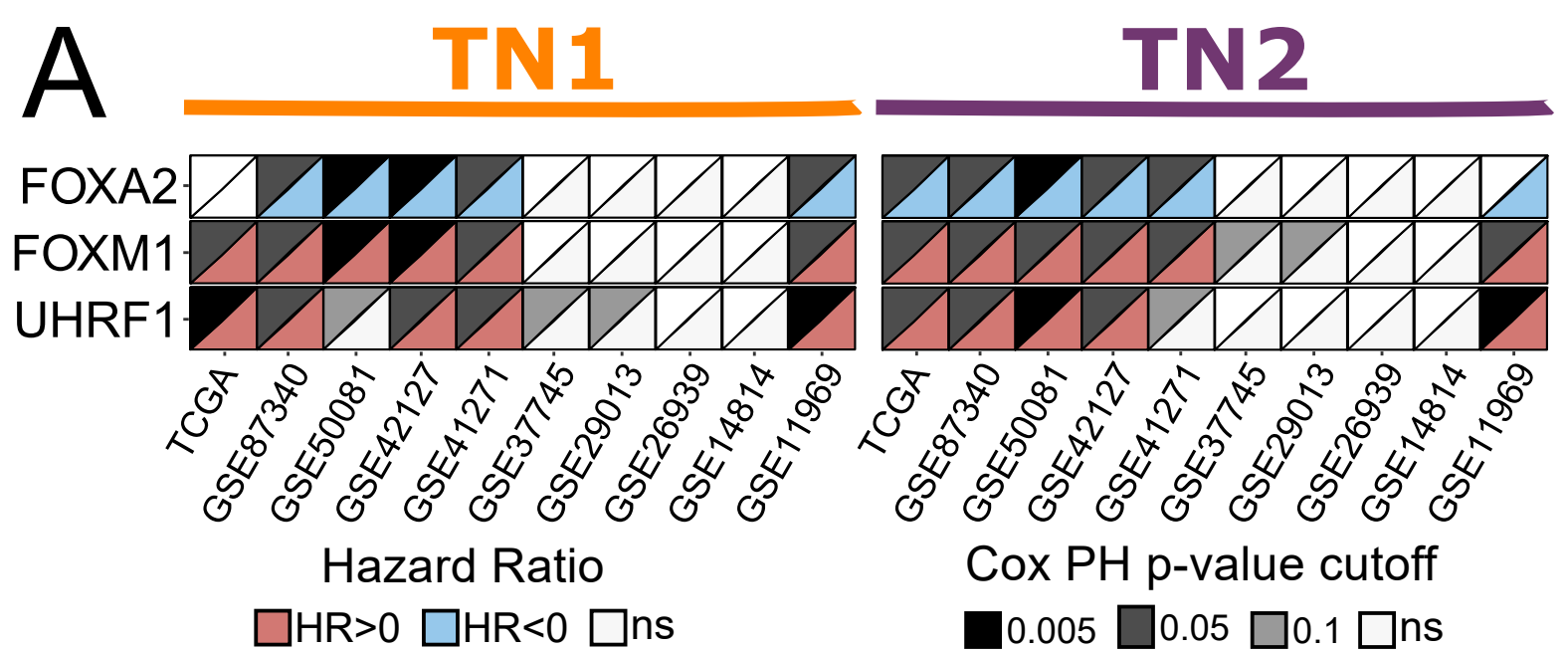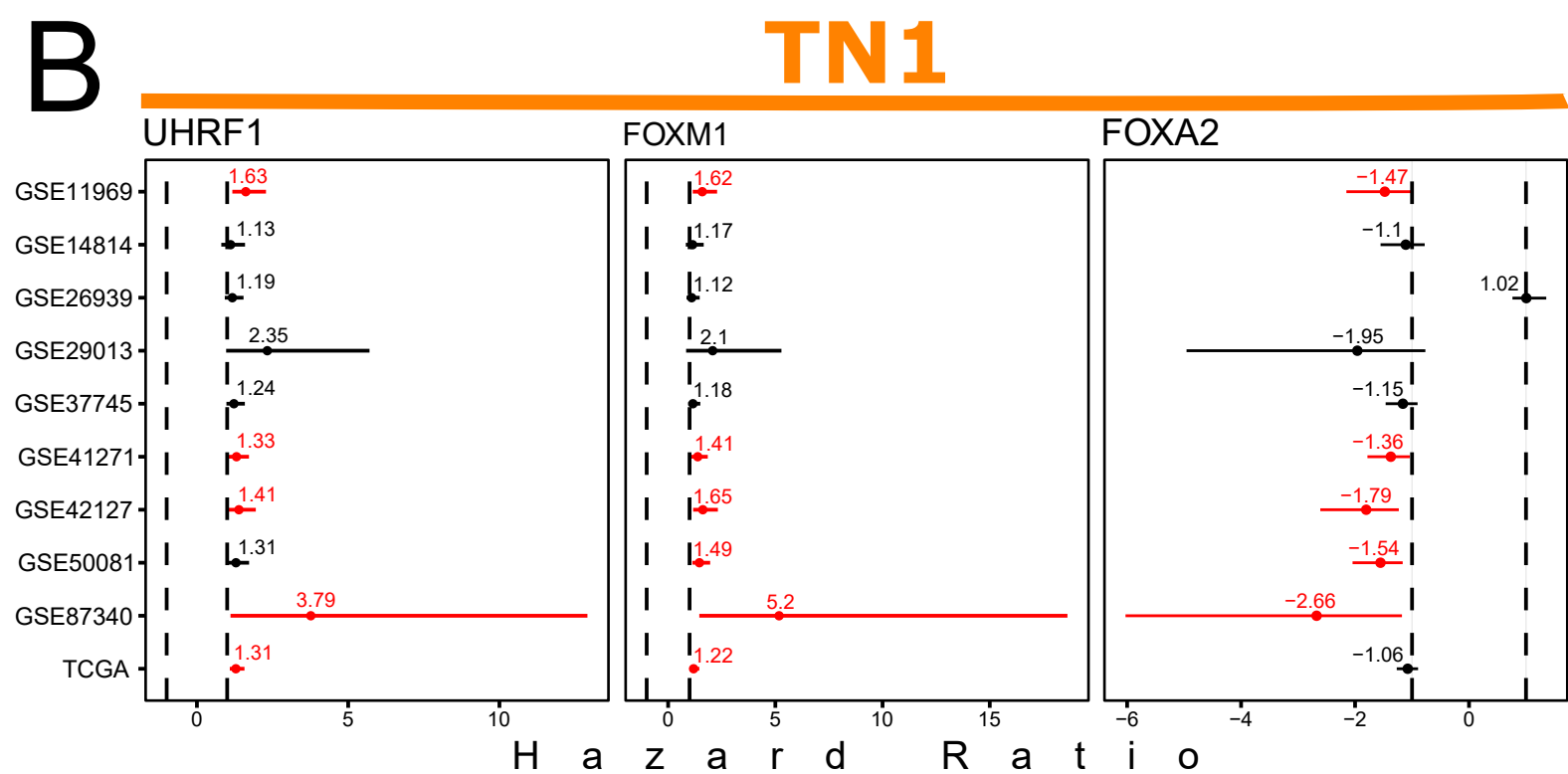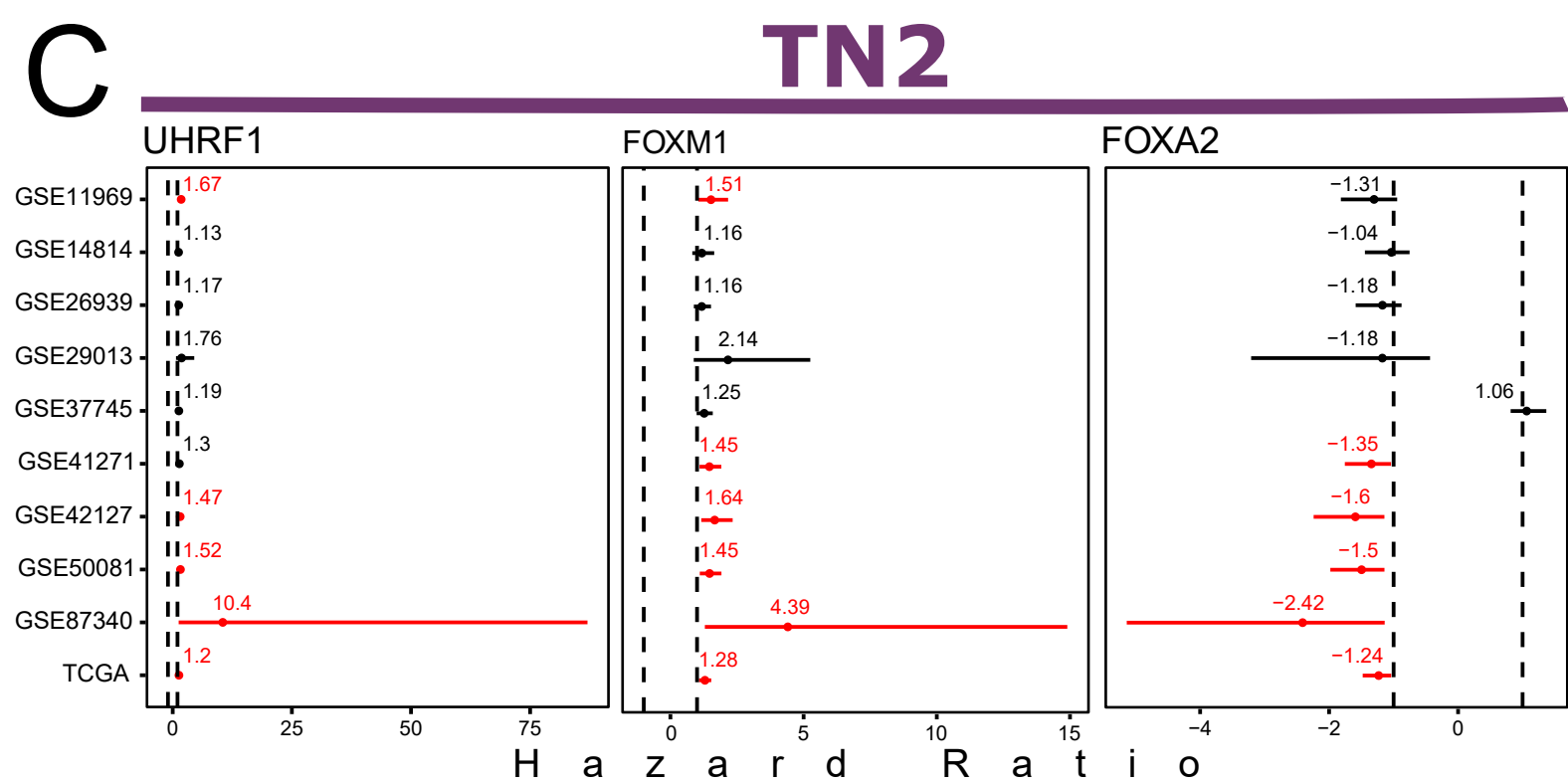

Supplement: Supplementary file 3 [file CAM4-8-6717-s003.pdf]

A

TN1

Number of Significant  
Cox Studies

○ [0-1]  
○ [2-4]  
● 5  
● 6  
● 7

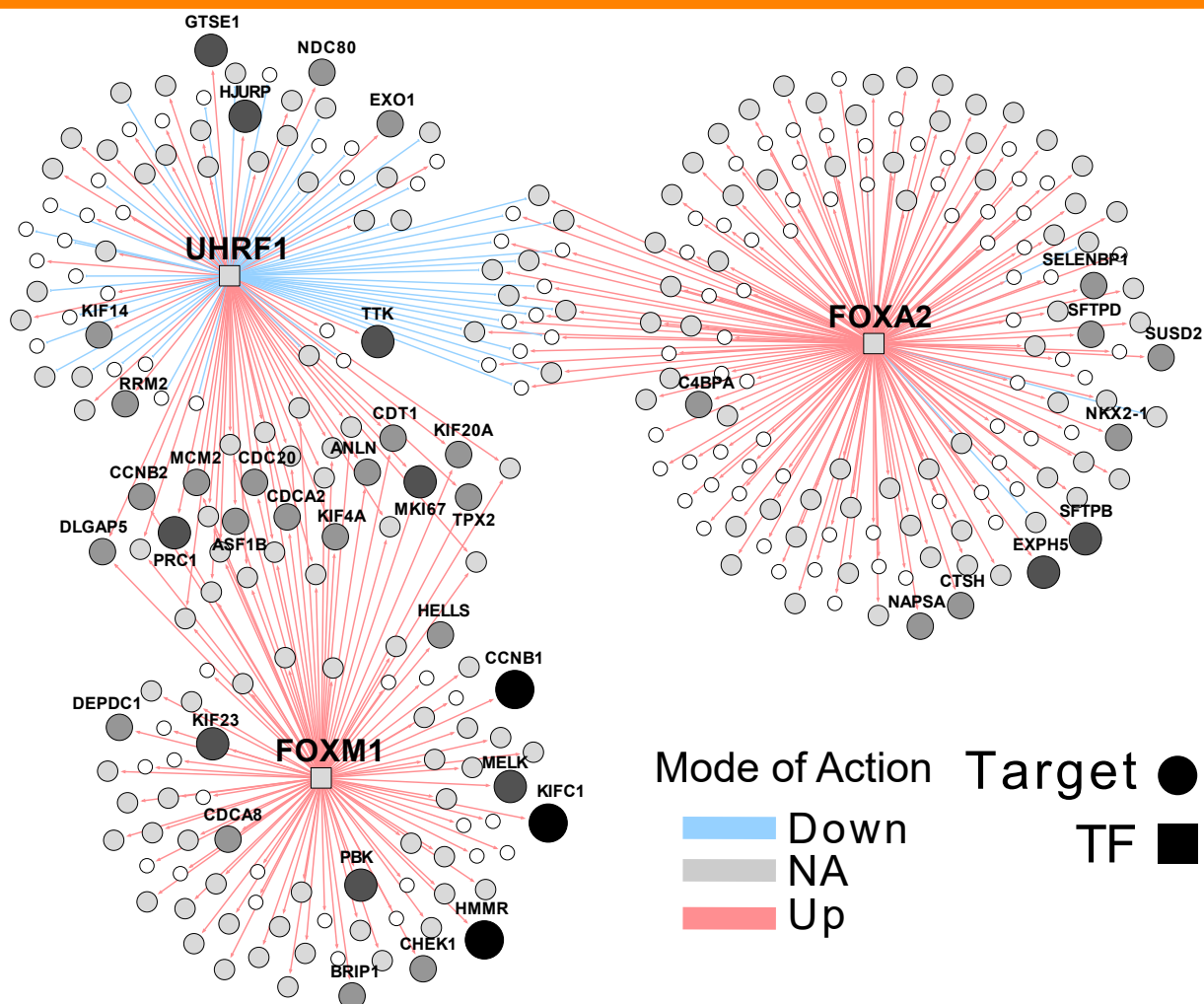

B

TN2

Number of Significant  
Cox Studies

○ [0-1]  
○ [2-4]  
● 5  
● 6  
● 7

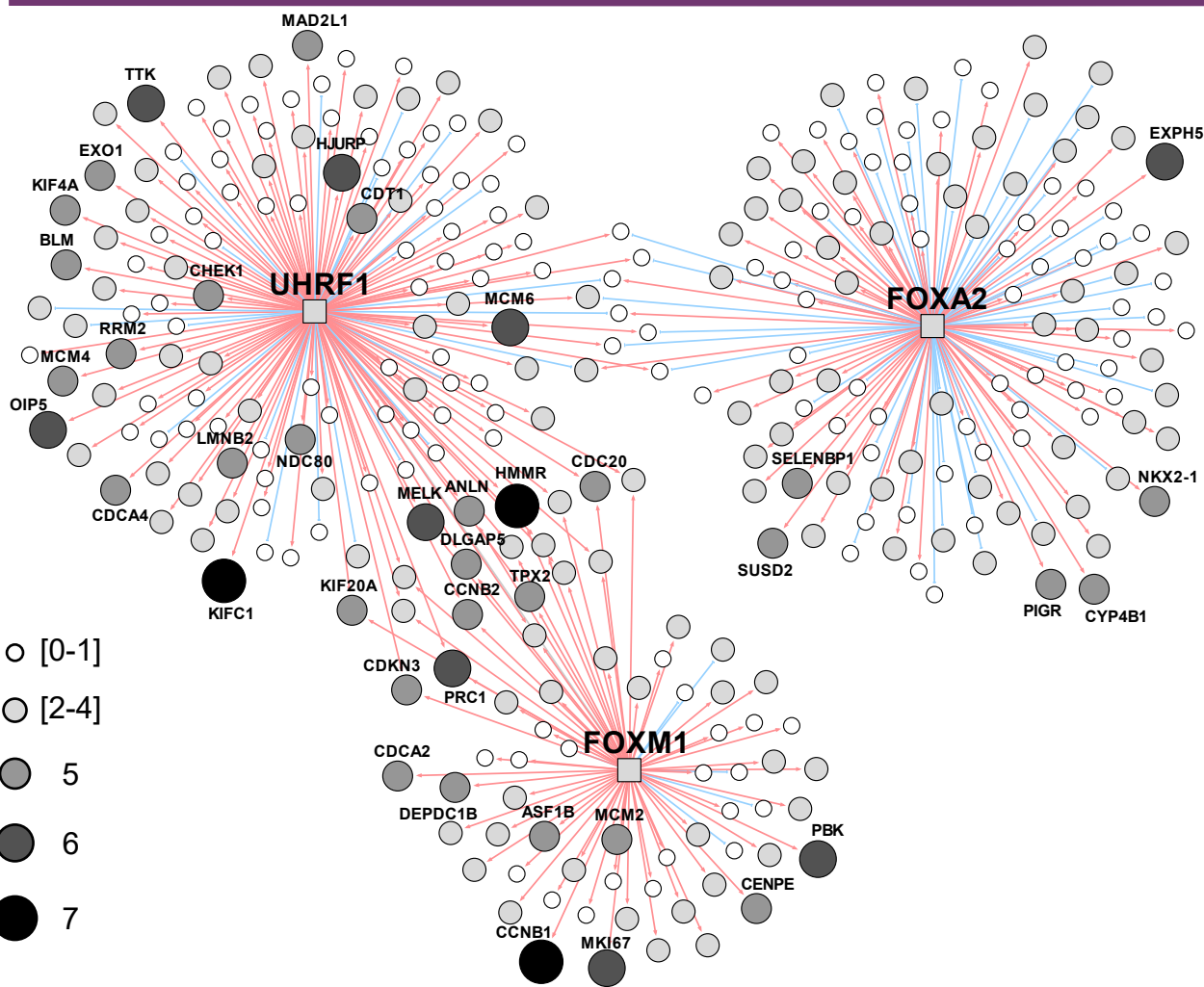

Supplement: Supplementary file 4 [file CAM4-8-6717-s004.pdf]
